# Decriminalizing suicide attempt in the 21st century: an examination of suicide rates in countries that penalize suicide, a critical review

**DOI:** 10.1186/s12888-022-04060-5

**Published:** 2022-06-23

**Authors:** Bob Lew, David Lester, Feisul Idzwan Mustapha, Paul Yip, Ying-Yeh Chen, Ravivarma Rao Panirselvam, Astrid Sinarti Hassan, Serena In, Lai Fong Chan, Norhayati Ibrahim, Caryn Mei Hsien Chan, Ching Sin Siau

**Affiliations:** 1 Australian Institute for Suicide Research and Prevention, School of Applied Psychology, Griffith University, Brisbane, Queensland Australia; 2 Department of Psychology, Stockton University, Galloway, NJ USA; 3 Non-Communicable Diseases Section, Disease Control Division, Ministry of Health, Malaysia, Putrajaya, Malaysia; 4 Department of Social Work and Social Administration, University of Hong Kong, Hong Kong, SAR China; 5 Center for Suicide Research and Prevention, University of Hong Kong, Hong Kong, SAR China; 6 Taipei City Psychiatric Centre, Taipei City Hospital, Taipei, Taiwan; 7 Institute of Public Health and Department of Public Health, National Yang-Ming University, Taipei, Taiwan; 8 Department of Psychiatry and Mental Health, Hospital Miri, Miri, Sarawak Malaysia; 9 Department of Medical Ethics and Law, Faculty of Medicine, Universiti Teknologi MARA, Shah Alam, Selangor Malaysia; 10 Department of Psychology, School of Medicine, International Medical University, Kuala Lumpur, Malaysia; 11 Department of Psychiatry, Faculty of Medicine, Universiti Kebangsaan Malaysia Medical Centre, Kuala Lumpur, Malaysia; 12 Faculty of Health Sciences, Universiti Kebangsaan Malaysia, Jalan Raja Muda Abdul Aziz, 50300 Kuala Lumpur, Malaysia

**Keywords:** Decriminalization, Criminalization, Suicide

## Abstract

**Background:**

Decriminalizing suicide may decrease overall suicide rates because then individuals who are at risk of suicide would be more willing to seek help from the community and from mental health professionals, therefore enabling early interventions for preventing suicidality. We aimed to examine the suicide trends over the last 20 years in 20 countries that still criminalize attempted suicide, and to compare the suicide rates of these 20 countries against the global average suicide rate and to a comparison sample of 20 countries that do not criminalize suicide, matched according to region and majority religion.

**Methods:**

Age-standardized suicide rates were extracted from the WHO Global Health Estimates, available for the period 2000-2019. Population data were extracted from the World Bank. We analyzed only countries which criminalize attempted suicide under its criminal justice system. Countries were further categorized according to their membership in the Commonwealth of Nations and countries in Africa. Countries from the same region and with the same majority religion were chosen as a matching group. Joinpoint analysis was used to compare the trends of the two groups with the global average.

**Results:**

Based on the 2019 WHO Global Health Estimates data, there is a large range in the suicide rates of the countries that criminalize attempted suicide, from 2.5 (Brunei) to 40.9 (Guyana) per 100,000 population. The mean suicide rate was 8.3 (Standard Deviation = 10.6). Out of the 20 countries, seven have suicide rates higher than the global average, covering a total population of about 387.3 million. Of these seven countries, five are in the African region. The other thirteen countries have suicide rates between 2.5 to 8.2. Mean scores of the countries which criminalized attempted suicide was lower than the global average and 20 comparison countries over the 20 years, but average annual percentage in the decrease of suicide was greater for countries in which attempted suicide was not criminalized.

**Conclusions:**

Based on our review, there was no substantial evidence here to indicate that countries which criminalized attempted suicide had consistently lower suicide rates compared to the global average. There is a need to acknowledge that the currently available evidence is inadequate to definitively claim that criminalizing suicide is beneficial or harmful for the reduction of suicide rate for the entire populations. Future studies should continue to evaluate the unique effects of decriminalizing attempted suicide while controlling for other key associated factors.

## Background

Suicide remains a major public health problem worldwide. The World Health Organization (WHO) estimates that there were over 700,000 deaths from suicide in the world in 2019, with an estimated suicide rate of 9.0 per 100,000 per year [1]. Suicide rate is defined as the number of deaths which are intentionally self inflicted per 100,000 people. Each suicide death leaves an estimated 135 other individuals exposed to the suicide [2]. Reducing the global suicide mortality rate by one third by 2030 is the sole target for the mental health field as outlined by the United Nations’ Sustainable Development Goals (SDGs) and the WHO’s Comprehensive Mental Health Action Plan 2013-2030 [3]. The WHO’s 13th General Program of Work 2019–2023 includes the same indicator, aiming for a reduction of 15% by 2023 [4].

In recent years, the world has seen changes in suicide rates. Overall, global suicide rates appear to be declining, but this is primarily a result of declines in suicide in lower- and middle-income countries, for example China and India [5]. China does not criminalize attempted suicide, while India decriminalized attempted suicide in 2017. The suicide rate in China declined from 14.9 in 2000 to 6.7 in 2019 with a decrease of 55.0% [6]. Similarly, the suicide rate in India declined from 19.1 to 12.9 during the same period with a decrease of 32.5%. The global average suicide rate declined from 14.0 to 9.0 in the similar period, totalling a decrease of 33.7%. Yet, other countries have witnessed increasing suicide rates, while it has been argued that suicide prevention programs are ineffective [7].

In twenty countries, attempted suicide is classified as a crime with accompanying punishment. However, in recent years, there has been a move in various countries to decriminalize attempted suicide, and the question arises of whether decriminalization of suicide attempts will result in an increase in suicidal behaviors. For example, since the introduction of the Suicide Act of 1961 in the United Kingdom, decriminalization was not followed by an increase in suicide rates. This is in contrast to Sweden, where suicide rates rose after suicide prohibition laws were repealed. It was thought that medical and police practise did not alter overnight, and the law had already been poorly adhered before 1961 in the UK [8].

It can be argued that decriminalizing suicide attempts may increase the suicide rate since there would no longer be any legal deterrent although this assumption has been refuted [9]. However, what we fear to be a worse outcome is that criminalizing suicide attempts may actually encourage people to use more lethal methods to end their life, instead of surviving their suicide attempt only to face imprisonment or having to go through the court process, on top of all the other existing societal stigma commonly faced thereafter. On the contrary, official suicide rates may unfortunately increase due to lower rates of under-reporting. Yet, in the long run, decriminalizing suicide may decrease overall suicide rates because then individuals who are at risk of suicide would be more willing to seek help from the community and from mental health professionals, therefore enabling early interventions for preventing suicidality [9].

To have a holistic picture of this issue, one must investigate the origin of classifying a suicide attempt as a criminal act. Suicide was considered a crime in England and Wales until 1961, as previously mentioned [8]. Nonetheless, the United Kingdom (UK) was one of the latest European countries to decriminalize attempted suicide, and the delay in English decriminalization is due to the structure of English common law. In 1959, the Church of England expressed worry that decriminalizing suicide would obviously imply “aiding and abetting suicide” [8]. As a result, suicidal behaviour still carries a heavy stigma. Despite the fact that data from other nations do not appear to be totally consistent, it appears that decriminalization was delayed in those countries impacted by English common law. Some speculated that the delay was due to the fact that common law, as opposed to written law, determines the subsequent judgement, despite the fact that public opinion had long before evolved, with a greater tolerance and softening of attitudes against suicide [10].

Moreover, the lengthy history of suicide as a criminal offence may still have a substantial contemporary effect on how it is regarded and conceptualized in today’s society, despite the fact that criminal law has a strong religious foundation. Suicide thus became a crime, according to another interpretation. As the law evolved, attempted suicide was classified as a misdemeanour, a low-level crime punishable by a fine or a brief jail sentence [11]. Despite the fact that suicide is no longer a criminal offence in Western countries, such as Australia in 1958, the United Kingdom (UK) in 1961, and Ireland in 1993, the lengthy history of suicide in these countries raises an essential question: how does public perception and stigmatization essentially influence actual suicide rates? [10].

History also reveals that the major religions of the world have classified suicide as a sin or as against the tenets of the religion. Most countries with a dominant religion have incorporated this moral stance into their initially unwritten and subsequently written laws. For example, some Christian denominations denied people who died by suicide the dignity of burial in cemeteries. Initially, Great Britain criminalized suicide and introduced it to many other Commonwealth countries. In fact, out of 20 countries which still presently criminalize attempted suicide, 15 are member states of the Commonwealth of Nations whereas 9 are from the African region. Since 1961, however, England has decriminalized attempted suicide.

The adoption of the Suicide Act gained attention from the UK’s Ministry of Health, who advised that all suicide attempters should instead receive psychiatric assistance and attention, based on recommendations by the British Medical Association. Thus, suicide took on a new meaning by the end of the nineteenth century. Suicide should not be vilified, but rather fostered as part of a rehabilitative system that is supported with a new set of rules and governing structures. Marsh in his book believes that suicide should be treated as a mental health issue [12]. Similarly, other countries that have also decriminalized attempted suicide include Sweden (1864), Finland (1910), New Zealand (1961), Hong Kong (1967), Canada (1972), Ireland (1993), Sri Lanka (1998), India (2017), and Singapore (2020) [12].

To answer the concern of whether decriminalizing attempted suicide would have an impact on the suicide rate, Lester [9] studied the impact of decriminalizing attempted suicide in seven countries: Canada, England and Wales, Finland, Hong Kong, Ireland, New Zealand and Sweden. The suicide rates in the five years prior to decriminalization and in the five years afterwards in these countries were compared. The mean suicide rate rose from 9.66 per 100,000 per year initially to 11.24 per 100,000 afterwards, a statistically significant increase.

Globally, efforts to decriminalize attempted suicide are ongoing in many countries. Despite the fact that many western countries have repealed criminalization, only several countries in Southeast Asia have repealed or are in the process of repealing criminalization of suicidal attempts. Indonesia, for example, does not consider suicide to be a crime. Some countries that adopt the Indian Penal Code, including Sri Lanka and Singapore, have since repealed criminalization as well [13]. Previously prior to its decriminalization, a person who attempted suicide in Singapore could face up to a year in prison. However, suicide attempts are still punishable in several African countries. Moreover, in North Korea, suicide is criminalized as a deterrent, with the family and relatives of suicide victims being punished as a kind of collective punishment [14].

Furthermore, Kahn and Lester [15, 16] documented the efforts to decriminalize attempted suicide in Ghana, India, and Singapore, noting that the pressure for this came from the Supreme Court in India, the legislature in Singapore, and non-governmental organizations (NGOs) and healthcare professionals in Ghana. The major reasons for this move included honouring human rights and the removal of the stigma of suicide so that individuals at risk of suicide will be more likely to seek professional help for their distress.

Thus, the aims of the present paper are: (1) to examine the suicide trends over the last 20 years in 20 countries that still criminalize suicide attempt, and (2) to compare the suicide rates of these 20 countries with 20 other countries that decriminalized suicide (matched by region and majority religion) and the global average suicide rate. We are interested to find out whether these countries that have consistently criminalized suicide attempts will have a lower suicide rate compared to the 20 comparison countries that decriminalized suicide and the global average.

## Method

All methods, where applicable, were performed according to the Declaration of Helsinki to ensure ethically conducted research.

### Data source

Age-standardized suicide rates were extracted from the WHO Global Health Estimates, available for the period 2000-2019 [1]. Population data were extracted from the World Bank [17].

### Criteria for inclusion and exclusion of countries in the study

Countries which were listed as criminalizing attempted suicide through the common or civil law by the United for Global Mental Health [18] from the document *Decriminalising Suicide: Saving Lives, Reducing Stigma* were included. As some of the laws involving Islamic and Syariah elements were not clear, this paper will analyze only countries which criminalize attempted suicide under its criminal justice system and will not include the 20 Muslim countries listed by Global Health Estimates in which attempted suicide is prosecuted under various provisions of Syariah laws instead.

Countries were further categorized according to their membership in the Commonwealth of Nations and countries in Africa. We have created the category of Commonwealth of Nations as there were 15 member states which criminalized attempted suicide.

The same number of countries that did not criminalize attempted suicide was chosen, matched according to region and majority religion, for comparison with the countries that criminalized attempted suicide.

### Statistical analysis

We calculated the mean scores of the age-standardized suicide rates of the 20 countries with and without a law criminalizing attempted suicide for each year, from 2000 to 2019. Using the mean scores obtained, Joinpoint regression was used to identify the occurrence of statistically significant changes in trend for countries with and without a law criminalizing attempted suicide. We used the average annual percentage change (AAPC) to examine the overall change, and the Annual Percentage Change (APC) to examine the annual change in age-standardized suicide rates during 2000-2019, with 95% confidence intervals (95% CIs). A *p*-value of less than 0.05 was deemed statistically significant for all two-tailed tests. Joinpoint version 4.9.0.1 was used to conduct the analyses.

## Results

### Characteristics of countries that criminalized attempted suicide

Fifteen out of the 20 countries that criminalize attempted suicide are Commonwealth countries, comprising 844.4 million population and represent about 86.8% of the total population of these 20 countries. Of the 20 countries wih a 972.6 million population, nine are in the African region with a total population of about 486.5 million representing about 50.0% of the total population.

**Fig. 1:**
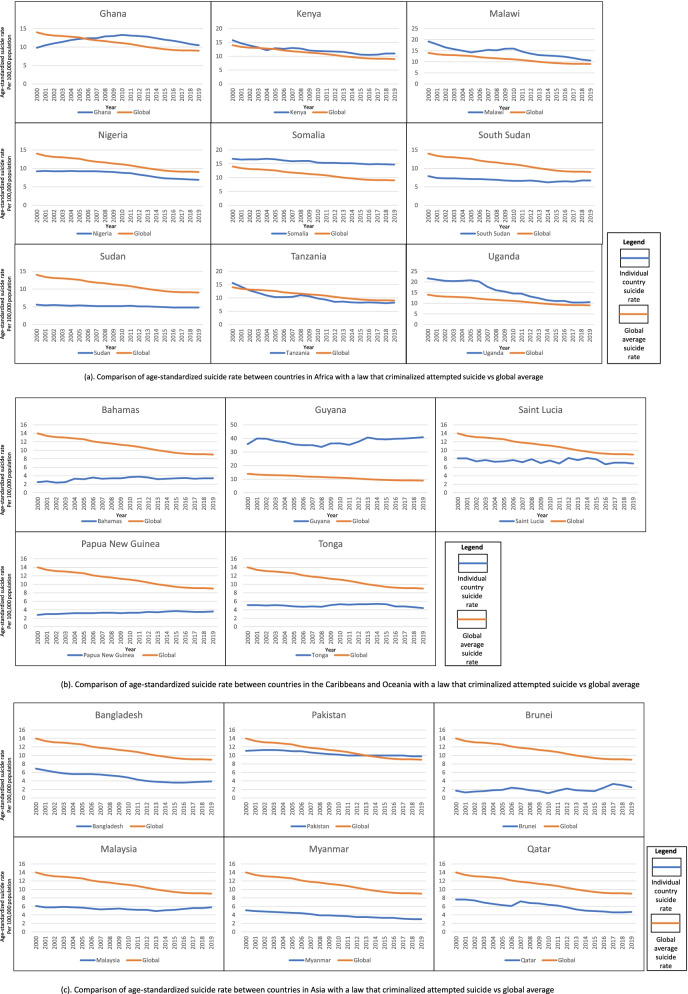
**a** Comparison of age-standardized suicide rate between countries in Africa with a law that criminalized attempted suicide vs global average. **b** Comparison of age-standardized suicide rate between countries in the Caribbeans and Oceania with a law that criminalized attempted suicide vs global average. **c** Comparison of age-standardized suicide rate between countries in Asia with a law that criminalized attempted suicide vs global average

Based on the 2019 Global Health Estimates data [1], there is a large range in the suicide rates of the countries that criminalize attempted suicide, from 2.5 (Brunei) to 40.9 (Guyana). The mean suicide rate was 8.3 (Standard Deviation = 10.6) per 100,000 population. Out of the 20 countries, seven have suicide rates higher than the global average, covering a total population of about 387.3 million. Of these seven countries, five are in the African region. The rest have suicide rates between 2.5 to 8.2 per 100,000 population. Five of the countries with laws against attempted suicide recorded an overall increase in the age-standardized suicide rates in 2000-2019. This includes Ghana (8.1%), Guyana (14.3%), Guinea (26.1%), Bahamas (33.2%) and Brunei (47.7%), whilst the rest recorded a decrease in suicide rates (Table 1) (Fig. 1).

**Table 1:** Characteristics of 20 Countries with laws against attempted suicide and comparison countries

Countries		UN Region/sub-region [19]	Commonwealth membership [20]	Majority religion [21]	Comparison country
1.	Ghana	Africa	Yes	Christianity	Namibia
2.	Kenya	Africa	Yes	Christianity	Mozambique
3.	Malawi	Africa	Yes	Christianity	Zambia
4.	Nigeria	Africa	Yes	Christianity	Cameroon
5.	Somalia	Africa	No	Muslim	Chad
6.	South Sudan	Africa	No	Christianity	DR Congo
7.	Sudan	Africa	No	Muslim	Central African Republic
8.	Tanzania	Africa	Yes	Christianity	Rwanda
9.	Uganda	Africa	Yes	Christianity	Botswana
10.	Bahamas	Caribbean	Yes	Christianity	Belize
11.	Guyana	Caribbean	Yes	Christianity	Trinidad and Tobago
12.	Saint Lucia	Caribbean	Yes	Christianity [22]	Barbados
13.	Papua New Guinea	Oceania	Yes	Christianity	Fiji
14.	Tonga	Oceania	Yes	Christianity	Kiribati
15.	Bangladesh	Asia	Yes	Muslim	Maldives
16.	Pakistan	Asia	Yes	Muslim	Afghanistan
17.	Brunei	Asia	Yes	Muslim	Oman
18.	Malaysia	Asia	Yes	Muslim	Indonesia
19.	Myanmar	Asia	No	Buddhism	Thailand
20.	Qatar	Asia	No	Muslim	Bahrain

Source: United Nations [20]. Commonwealth Secretariat (2022) [21]. World Populaton Review (2022) [22]. Encyclopaedia Britannica (2022) [23]

Mean scores of age-standardized suicide rates during the past 20 years for countries that had a law criminalizing attempted suicide were lower than the mean scores of countries that did not have a law which criminalized attempted suicide as well as the global average during 2000 to 2019 (Tables 2 and 3).

**Table 2:** Age-standardized suicide rates per 100,000 population^a^ [20] in 2000-2019 in countries with laws against attempted suicide

	UN Region/sub-region^b^	Countries	2000	2001	2002	2003	2004	2005	2006	2007	2008	2009	2010	2011	2012	2013	2014	2015	2016	2017	2018	2019	% Change
1	Africa	Ghana	9.8	10.5	11.0	11.4	11.9	12.2	12.4	12.4	12.9	13.0	13.3	13.1	13.0	12.8	12.4	12.0	11.7	11.3	10.8	10.5	8.1%
2	Africa	Kenya	15.8	14.7	13.9	13.2	12.2	12.9	12.7	13.0	12.8	12.1	11.9	11.8	11.7	11.6	11.1	10.6	10.5	10.6	11.0	11.0	−30.4%
3	Africa	Malawi	19.2	17.9	16.5	15.7	15.0	14.3	14.8	15.4	15.2	15.9	16.0	14.6	13.7	13.0	12.8	12.6	12.3	11.7	11.0	10.6	−45.0%
4	Africa	Nigeria	9.2	9.3	9.2	9.2	9.3	9.2	9.2	9.2	9.1	9.0	8.8	8.7	8.3	8.0	7.6	7.3	7.2	7.1	7.0	6.9	−25.2%
5	Africa	Somalia	16.8	16.5	16.6	16.6	16.8	16.6	16.2	15.9	16.0	16.0	15.4	15.3	15.3	15.2	15.2	15.0	14.8	14.9	14.8	14.7	−12.6%
6	Africa	South Sudan	7.9	7.4	7.3	7.3	7.2	7.1	7.1	7.0	6.9	6.7	6.6	6.6	6.7	6.5	6.2	6.4	6.5	6.4	6.7	6.7	−15.2%
7	Africa	Sudan	5.6	5.4	5.5	5.4	5.3	5.4	5.3	5.2	5.2	5.2	5.2	5.3	5.1	5.1	5.0	4.9	4.8	4.8	4.8	4.8	−14.4%
8	Africa	Tanzania	15.6	14.2	12.8	11.9	10.9	10.3	10.3	10.4	11.0	10.6	9.8	9.3	8.5	8.6	8.3	8.2	8.3	8.2	8.0	8.2	−47.6%
9	Africa	Uganda	21.7	21.1	20.5	20.4	20.5	20.8	20.2	17.7	16.1	15.5	14.6	14.5	13.2	12.4	11.4	11.0	11.1	10.3	10.3	10.5	− 51.7%
10	Caribbean	Bahamas	2.5	2.7	2.4	2.5	3.3	3.2	3.6	3.3	3.4	3.4	3.7	3.8	3.6	3.2	3.3	3.4	3.5	3.3	3.4	3.4	33.2%
11	Caribbean	Guyana	35.8	39.9	39.8	38.1	37.3	35.6	35.0	35.1	33.7	36.3	36.4	35.3	37.6	40.7	39.5	39.3	39.7	39.9	40.4	40.9	14.3%
12	Caribbean	Saint Lucia	8.1	8.1	7.4	7.7	7.3	7.4	7.7	7.2	7.9	7.0	7.6	6.9	8.2	7.7	8.2	7.9	6.7	7.1	7.1	6.9	−15.2%
13	Oceania	Papua New Guinea	2.8	3.0	3.0	3.1	3.2	3.2	3.2	3.3	3.3	3.2	3.3	3.3	3.5	3.4	3.6	3.7	3.6	3.5	3.5	3.6	26.1%
14	Oceania	Tonga	5.1	5.1	5.0	5.1	5.0	4.8	4.7	4.8	4.7	5.1	5.3	5.2	5.3	5.3	5.4	5.3	4.8	4.8	4.6	4.4	−14.8%
15	Asia	Bangladesh	6.9	6.5	6.1	5.8	5.6	5.6	5.6	5.5	5.3	5.1	4.8	4.3	4.0	3.8	3.7	3.6	3.6	3.7	3.8	3.9	−44.0%
16	Asia	Pakistan	11.1	11.2	11.3	11.3	11.2	11.0	11.0	10.7	10.5	10.3	10.2	10.0	10.0	10.0	10.0	10.0	10.0	10.0	9.8	9.8	−12.0%
17	Asia	Brunei	1.7	1.3	1.5	1.6	1.8	1.9	2.4	2.2	1.8	1.6	1.1	1.7	2.2	1.8	1.7	1.6	2.4	3.3	3.0	2.5	47.7%
18	Asia	Malaysia	6.1	5.8	5.8	5.9	5.8	5.7	5.5	5.3	5.4	5.5	5.3	5.2	5.2	4.9	5.1	5.2	5.4	5.6	5.6	5.8	−5.4%
19	Asia	Myanmar	5.1	4.9	4.8	4.7	4.6	4.5	4.4	4.2	3.9	3.9	3.8	3.7	3.5	3.5	3.4	3.3	3.3	3.1	3.0	3.0	−42.4%
20	Asia	Qatar	7.6	7.6	7.4	6.9	6.6	6.3	6.1	7.2	6.8	6.7	6.4	6.2	5.8	5.3	5.0	4.9	4.8	4.6	4.6	4.7	−38.8%
	Mean score		10.7	10.7	10.4	10.2	10.0	9.9	9.9	9.8	9.6	9.6	9.5	9.2	9.2	9.1	8.9	8.8	8.8	8.7	8.7	8.6	
	Standard Error		1.83	1.95	1.93	1.85	1.80	1.74	1.70	1.68	1.61	1.72	1.72	1.66	1.73	1.87	1.81	1.80	1.81	1.81	1.83	1.85	
	Global	Global	14.0	13.4	13.1	13.0	12.8	12.6	12.1	11.8	11.6	11.3	11.1	10.8	10.4	10.0	9.7	9.4	9.2	9.1	9.1	9.0	−36.1%

Source: ^a^WHO Global Health Estimates (2021) [1]. ^b^United Nations (2021) [19]

**Table 3:** Age-standardized suicide rates per 100,000 population ^a^ [1] in 2000-2019 in countries without laws against attempted suicide

	UN Region/sub-Region b[19]	Countries	2000	2001	2002	2003	2004	2005	2006	2007	2008	2009	2010	2011	2012	2013	2014	2015	2016	2017	2018	2019	% Change
1	Africa	Namibia	27.5	26.7	26.3	25.5	23.4	24.7	24.5	23.7	19.7	18.4	18.0	16.1	14.5	14.4	15.3	16.3	14.9	15.2	13.7	13.5	−50.9%
2	Africa	Mozambique	20.9	20.8	20.8	20.9	21.1	21.4	21.6	21.8	23.1	24.6	25.3	25.9	26.5	26.0	24.7	24.0	23.4	23.1	23.2	23.2	10.9%
3	Africa	Zambia	24.0	22.7	22.3	21.5	22.5	22.1	21.8	19.8	19.0	19.0	19.7	19.8	18.5	17.9	17.7	17.3	16.7	16.7	15.9	14.4	−40.0%
4	Africa	Cameroon	19.1	19.4	19.2	19.5	19.4	20.6	20.9	20.4	20.1	20.0	19.5	19.4	19.2	18.7	17.9	17.1	16.6	16.2	15.9	15.9	−16.7%
5	Africa	Chad	15.7	15.8	15.8	16.0	15.7	15.6	15.6	15.9	15.7	15.8	16.0	15.9	15.6	15.1	14.6	14.0	13.7	13.8	13.6	13.2	−15.8%
6	Africa	DR Congo	14.5	14.4	14.2	14.4	14.2	14.1	13.9	13.4	13.4	13.6	13.5	13.3	13.0	12.8	12.6	12.3	12.1	12.0	12.4	12.4	−14.4%
7	Africa	Central African Republic	32.5	30.7	30.4	29.1	28.1	27.2	28.1	27.7	27.3	28.5	28.1	27.6	26.6	27.0	27.6	26.3	25.3	25.7	23.7	23.0	−29.4%
8	Africa	Rwanda	25.6	23.0	20.7	18.4	16.2	15.0	14.3	13.7	12.9	12.1	11.5	10.9	10.6	10.1	10.1	9.9	9.7	9.6	9.5	9.5	− 63.0%
9	Africa	Botswana	46.3	44.7	42.2	40.3	40.9	43.4	40.9	37.5	36.1	32.1	31.2	29.1	29.0	27.8	25.4	24.6	22.9	22.3	22.1	20.2	− 56.3%
10	Caribbean	Belize	10.0	8.2	8.1	8.2	8.8	7.1	7.2	6.7	5.7	7.5	8.2	7.9	7.4	6.8	6.9	6.9	6.7	7.7	7.2	7.7	−23.6%
11	Caribbean	Trinidad and Tobago	16.2	16.1	14.9	12.9	12.6	12.1	10.7	10.5	12.8	10.4	10.6	9.2	8.9	10.1	9.8	9.5	9.2	9.0	8.5	8.3	−48.9%
12	Caribbean	Barbados	2.6	1.5	0.3	0.8	1.0	2.2	1.3	1.3	0.7	1.1	1.2	0.6	0.2	0.1	0.3	0.3	0.3	0.3	0.3	0.3	− 88.1%
13	Oceania	Fiji	11.7	11.4	11.0	10.5	10.0	10.2	9.9	10.1	10.2	10.2	10.1	10.3	10.5	10.5	10.3	10.0	10.0	9.9	9.7	9.5	−18.6%
14	Oceania	Kiribati	35.6	35.3	34.5	32.5	32.0	32.5	31.5	32.8	33.5	33.0	32.7	32.9	32.0	32.3	31.6	31.6	31.4	31.5	30.3	30.6	−14.3%
15	Asia	Maldives	5.3	5.1	4.7	4.6	3.8	3.8	3.8	3.6	3.5	3.3	3.1	2.9	2.9	2.8	2.9	3.0	3.2	3.1	2.9	2.8	−47.9%
16	Asia	Afghanistan	7.7	7.9	7.9	7.7	7.8	7.6	7.6	7.4	7.2	6.8	6.7	6.4	6.2	6.2	6.0	6.0	6.0	6.0	5.9	6.0	−22.7%
17	Asia	Oman	6.7	6.7	6.3	6.4	6.1	6.2	6.5	6.5	6.3	6.0	6.1	5.5	4.9	4.8	4.7	4.7	4.5	4.5	4.4	4.5	−33.4%
18	Asia	Indonesia	3.8	3.7	3.6	3.6	3.5	3.3	3.2	3.2	3.1	3.0	2.9	2.9	2.8	2.7	2.6	2.6	2.6	2.6	2.6	2.6	−32.7%
19	Asia	Thailand	11.6	10.5	10.5	10.2	9.9	8.5	8.3	7.7	7.4	7.3	7.3	7.2	7.0	6.6	6.9	7.2	7.6	7.5	7.9	8.0	− 31.4%
20	Asia	Bahrain	7.0	8.9	9.2	9.9	9.8	9.6	7.8	8.7	8.3	7.7	7.2	6.9	6.4	6.2	6.4	6.3	6.5	6.7	7.0	7.2	2.3%
	Mean score																						
	Standard Error																						
	Global	Global	14.0	13.4	13.1	13.0	12.8	12.6	12.1	11.8	11.6	11.3	11.1	10.8	10.4	10.0	9.7	9.4	9.2	9.1	9.1	9.0	−36.1%

Source: ^a^WHO Global Health Estimates (2021) [1]. ^b^United Nations (2021) [19]

**Fig. 2:**
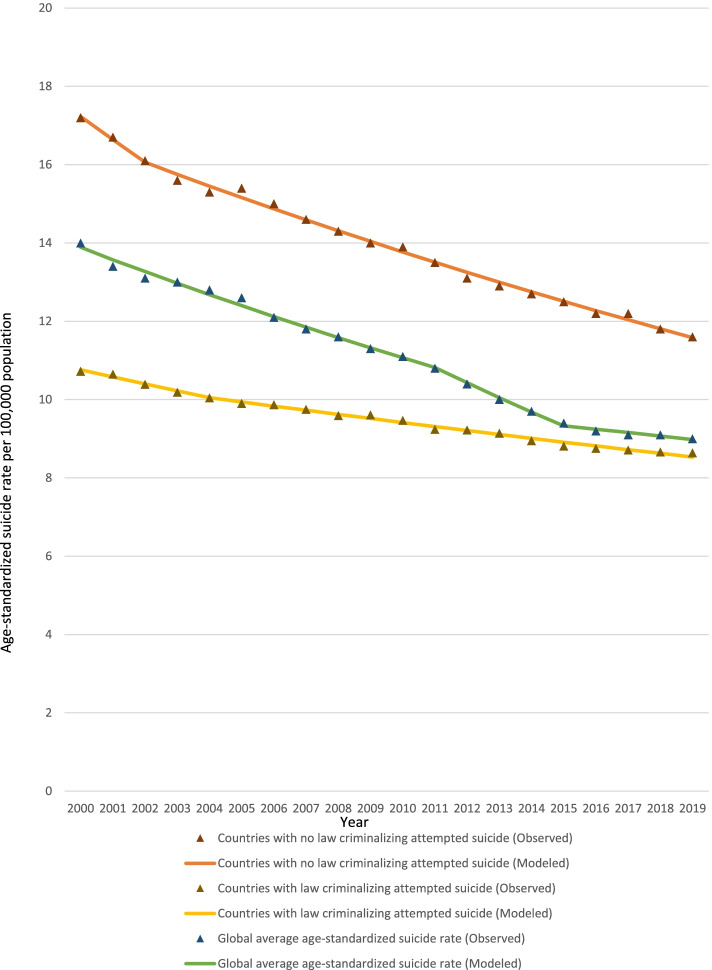
Age-standardized suicide rates in 2000-2019 in countries which criminalized attempted suicide and comparisons with countries which did not criminalize attempted suicide and the global average rate

The Joinpoint regression identified year 2004 as the Joinpoint for countries with a law criminalizing attempted suicide. Age-standardized suicide rates did significantly decline between 2000 and 2004 (APC: -1.7, 95% CI: − 2.3 to − 1.1, *p* <  0.001), and between 2004 and 2019 (APC: -1.1, 95% CI: − 1.2 to − 1.0, *p* <  0.001), with an AAPC of − 1.2 (95% CI: − 1.3 to − 1.1, *p* <  0.001). In comparison, for countries without a law that criminalized attempted suicide, the age-standardized suicide rates significantly declined between 2000 and 2002 (APC: -3.5, 95% CI: − 5.8 to − 1.1, *p* = 0.007), and between 2002 and 2019 (APC: -1.9, 95% CI: − 2.0 to − 1.8, *p* <  0.001), with an AAPC of − 2.1 (95% CI: − 2.3 to − 1.8, *p* <  0.001) (Table 4) (Fig. 2).

**Table 4:** Joinpoint analysis of age-standardized suicide rates in countries with and without a law that criminalized attempted suicide for 2000-2019

Countries criminalizing attempted suicide	**Segment**	**Lower Endpoint**	**Upper Endpoint**	**APC**	**Lower CI**	**Upper CI**	**Test Statistic (t)**	**Prob > |t|**
	1	2000	2004	−1.7*	−2.3	−1.1	−6.0	< 0.001
	2	2004	2019	−1.1*	−1.2	−1.0	−26.3	< 0.001
	**Segment**	**Lower Endpoint**	**Upper Endpoint**	**AAPC**	**Lower CI**	**Upper CI**	**Test Statistic (t)**	**Prob > |t|**
	Full Range	2000	2019	−1.2*	− 1.3	− 1.1	−17.8	< 0.05
Countries not criminalizing attempted suicide	**Segment**	**Lower Endpoint**	**Upper Endpoint**	**APC**	**Lower CI**	**Upper CI**	**Test Statistic (t)**	**Prob > |t|**
	1	2000	2002	−3.5*	−5.8	−1.1	−3.1	0.007
	2	2002	2019	−1.9*	−2.0	−1.8	−47.2	< 0.001
	**Segment**	**Lower Endpoint**	**Upper Endpoint**	**AAPC**	**Lower CI**	**Upper CI**	**Test Statistic (t)**	**Prob > |t|**
	Full Range	2000	2019	−2.1*	−2.3	−1.8	−16.7	< 0.05

*APC* Annual Percentage Change, *AAPC* Average Annual Percentage Change, *CI* Confidence Intervals

## Discussion

Based on our review, the mean scores of age-standardized suicide rates for countries that had a law criminalizing attempted suicide were lower than the mean scores of countries that did not criminalize it during the past 20 years and the global average. The results suggest that criminalizing suicide may have a protective effect against suicide as perhaps is the intention of authorities. However, the lower rates may also be due to underreporting and misclassification of suicide [19].

On the other hand, country-level comparisons with the global average revealed that there is a large range in the suicide rates of the countries that criminalize attempted suicide. Therefore, there was no sufficient evidence here to indicate that countries which criminalized attempted suicide had consistently lower suicide rates compared to the global average. Likewise, Mishara and Weisstub [20] found that the suicide rates of countries that criminalized attempted suicide were not consistently higher or lower than the global average. It should be noted, however, that Muslim countries that adhere to Syariah or Islamic law, where a suicide attempt is an offense punishable with jail sentences, have been found to have lower national suicide rates [21].

Lester [15] found that suicide rates increased after decriminalizing attempted suicide in Canada (1972) and Ireland (1993). Yet, suicide rates in Sri Lanka had since decreased after the decriminalization of attempted suicide in 1996, which may be associated with suicide prevention efforts implemented after the law was abolished [15, 22]. Many are Commonwealth countries which suggest the law was a leftover from the UK’s colonial past and from the lead that the UK has decriminalized its initial Suicide Act in 1960.

Based on the Joinpoint regression analyses, we found that countries which did not criminalize attempted suicide had a greater decrease in age-standardized suicide rates in comparison with countries that criminalized it. This was true even after matching the criminalizing and non-criminalizing countries according to region and majority religion (except Singapore, which was matched with Brunei due to its region, but not according to a similar majority religion). Perhaps among countries in which suicide attempt is not a crime, individuals who attempted were able to seek help for their mental and emotional conditions. Previous studies have shown that a previous suicide attempt is considered a high risk factor for future subsequent attempts, with one study recording 20.1% of suicide attempters reattempting within a twelve-month period of follow-up [23]. A study among Chinese undergraduate students showed that those with a lifetime history of a suicide attempt continued to report higher depression, hopelessness, and psychache in comparison to those without a suicide attempt history [24]. This suggests that suicide attempters need continuous support for their mental health, and an environment which encourage reporting a suicide attempt may increase the likelihood of help-seeking.

Therefore, decriminalizing attempted suicide should not be discounted as part of a national suicide prevention strategy [25]. While criminalizing suicide attempts may appear to have some deterrent effect against an individual choosing to attempt suicide, however, the extent of its effectiveness in preventing suicide depends largely on many other individual factors. On the one hand, the deterrence of being penalized for attempting suicide might cause certain individuals to rethink attempting suicide. On the other hand, criminalization might deter them from seeking help and this hinders early intervention for suicide prevention. There can be some complex interaction and offsetting the impact of each other.

Suicide is a multifactorial issue, often originating from immense psychological pain and hopelessness. One such model that is useful as a frame of reference is Turecki’s [26] biopsychosocial model for suicide risk which outlines the various factors that contribute to suicide risk, namely the biological, sociological, demographic, economic and environmental factors. These factors may influence any or all of the distal, developmental and proximal factors which are associated in turn with suicidal ideation, attempts and completed suicide. Therefore, there is a need to acknowledge that the currently available evidence is still inadequate to definitively claim that criminalizing attempted suicide is beneficial or harmful for the entire populations. Future studies should continue to evaluate the unique effects of decriminalizing attempted suicide while considering other factors that could also contribute to it. It certainly helps to destigmatize suicide and facilitate early identification and treatment.

If we were to achieve the WHO’s suicide reduction target [27], we must, therefore, consider the possibility that the means to this end may not lie in the legal domain of prosecuting suicide attempters. Rather, suicide prevention in the twenty-first century should follow evidence-based strategies such as those that the WHO has recommended to all countries and which can be implemented cross-culturally for effective suicide prevention. These include the need to limit access to the means for suicide, working with the media for responsible reporting of suicide, fostering socio-emotional life skills in adolescents, and early identification, assessment, management and follow-up for individuals who are affected by suicidal ideation and may start displaying suicidal behaviors. The way to this common goal is through the lens of compassionate humanity – the need to treat individuals who have experienced suicide attempt(s) as individuals in need of help rather than criminals in the court of law. One size does not fit all and decriminalizion is not the magic pill for suicide prevention.

Notably, this study has several limitations. Firstly, we did not attempt to conduct an in-depth analysis of the associations between severity of the penalty for attempting suicide in different countries with its suicide rates. Secondly, we had used region and majority religion as the criteria for matching 20 countries which did not criminalize attempted suicide. There is a possibility that comparisons with a different set of countries may yield different results.

An environment of fear and stigmatization may discourage people in need of help to be hesitant to seek help. Future studies should seek to examine the significant impact of decriminalizing suicide in decreasing stigma and encouraging help-seeking along with other public health campaigns in suicide prevention efforts should the data become available. It should, however, be considered that in developing nations, it may not be feasible to cater to population increases in help-seeking behaviour following suicide decriminalization should the number of available mental health professionals not increase commensurately. The access to mental health services to supporting suicidal people should be increased accordingly and this is best met as a concerted systemic collaboration between the government authorities, mental health industry, as well as, as the public.

## Data Availability

The datasets used and/or analysed during the current study are available from the WHO Global Health Estimates and are open access.
